# Wrapping culture plates with Parafilm M^®^ increases *Caenorhabditis elegans* growth

**DOI:** 10.1186/s13104-019-4854-3

**Published:** 2019-12-19

**Authors:** Jessica H. Shinn-Thomas, Sara E. Scanga, Patrick S. Spica, Hardik K. Nariya, Emra Klempic, Mary R. Brockett

**Affiliations:** 1 Department of Biology, Utica College, 1600 Burrstone Road, Utica, NY 13502 USA; 2 Division of Cardiac Surgery, University of Rochester Medical Center, 601 Elmwood Avenue, Rochester, NY 14642 USA; 3 Department of Microbiology and Immunology, Uniformed Services University, 4301 Jones Bridge Road, Bethesda, MD 20814 USA

**Keywords:** Nematode Growth Media, NGM, *C. elegans*, Parafilm, Larval growth

## Abstract

**Objective:**

Parafilm M^®^ is a moisture-resistant thermoplastic commonly used to seal Nematode Growth Media (NGM) agar plates on which the nematode *Caenorhabditis elegans* is cultured. This practice reduces media dehydration and microbial contamination. However, the effects on *C. elegans* individuals of placing this barrier between the external environment and the interior of the NGM plate are currently unknown. Our research aims to determine if this common practice engenders developmental changes, such as growth, that could subsequently and unintentionally alter experimental data. We compared the larval growth over 48 h of animals cultured on Parafilm-wrapped and unwrapped control NGM plates.

**Results:**

Wrapping culture plates with Parafilm significantly accelerated and increased larval growth, with a 0.87 μm/h increase in growth rate (~ 6%) and a 37.90 μm increase in the change in growth (Δgrowth; ~ 5%) over 48 h. Therefore, *C. elegans* investigators should be aware that wrapping their experimental cultures with Parafilm may result in statistically detectable changes in worm growth and possibly other developmental processes.

## Introduction

*Caenorhabditis elegans* (*C. elegans*), a microscopic and self-fertilizing hermaphroditic nematode, is an exceedingly well-established model organism [1]. It is used in diverse research areas ranging from environmental and natural history studies to biomedical and chemical research [1–5]. *C. elegans* individuals naturally colonize humid and nutrient-rich soil habitats such as compost piles, garden soil, and decomposing plant material. They coexist with and consume microorganisms including bacteria and microscopic eukaryotes [4, 6–10]. In the lab, they are typically cultured in Petri dishes on a nutrient rich agar media, Nematode Growth Media (NGM). The media is inoculated with an *Escherichia coli* (*E. coli*) food source, usually a uracil auxotroph mutant strain of *E. coli* (OP50; [11]). Unfortunately, these nutrient-rich laboratory cultures of *C. elegans* can easily succumb to microbial contamination despite careful use of sterile technique.

**Fig. 1:**
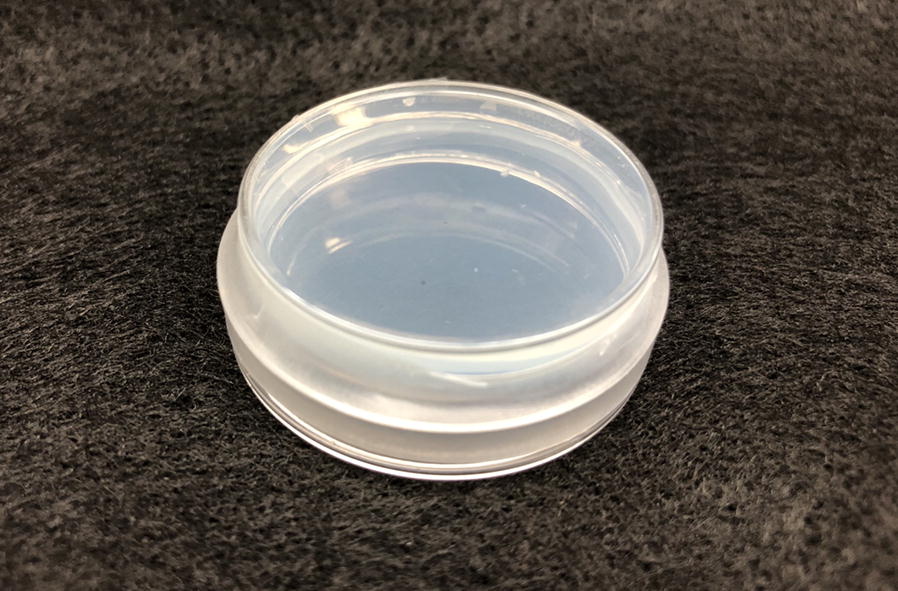
Parafilm M^®^ is commonly used to seal NGM culture plates. Parafilm wrapped one time around the circumference of a NGM culture plate

*Caenorhabditis elegans* researchers commonly wrap and seal their NGM agar culture plates with Parafilm M^®^ (Parafilm), which is a moisture-resistant thermoplastic tape used in research laboratories (Fig. 1), to avoid contamination and media dehydration. Despite the prevalence of this practice, it is unknown if Parafilm sealing causes any secondary effects to worm experiments. Given that the design of Petri plates allows for moisture and air exchange between the external and internal environments, the Parafilm might alter gas exchange and/or increase humidity in the culture environment under which the worms are developing compared to cultures not wrapped with Parafilm. Under laboratory conditions, *C. elegans* individuals may be unable to avoid changes to environmental conditions within the culture that result from Parafilm wrapping, and it is possible that these altered conditions may affect worm development.

In this study, we aimed to identify if sealing NGM culture plates with Parafilm altered larval growth. We found statistically significant, moderate increases in larval growth in worms cultured in plates wrapped with Parafilm compared to control worms. These results necessitate an awareness of *C. elegans* biologists to the unintended secondary effects this common lab practice could have on *C. elegans* biology and experimental datasets.

## Main text

### Materials and methods

#### Culture conditions

The Bristol wild-type *C. elegans* strain (N2) obtained from the *Caenorhabditis* Genetics Center (CGC) was grown in vented 35 mm plates (Tritech Research, Inc., #T3500) containing Nematode Growth Media with 2 mg/mL uracil (NGM). NGM was inoculated with the *E. coli* strain OP50 (CGC) grown in Miller’s Luria Broth (Fisher Scientific, #50-488-764). Worms were cultured at 20 **°**C ± 0.2 **°**C (Conviron I25L Growth Chamber), and standard culture conditions and methods were used [11, 12].

#### Larval growth

Larvae were synchronized at the L1 stage according to Brockett et al. [13]. L1 hermaphrodite worms were individually transferred at t = 0 h to NGM plates inoculated with 50 μL of the same overnight OP50 culture supplied ad libitum. Each plate was assigned to a treatment group using a random number table (n = 41 plates per group, each with one worm). Plates in the Parafilm treatment group were wrapped one time around the circumference of the plate with Parafilm™ M Wrapping Film (Fisher Scientific, #S37440), sealing the lid to the bottom of the Petri dish (Fig. 1). Control plates were not wrapped with Parafilm. There was no significant difference in median worm length between the Parafilm-treated and control worms at the time of transfer (Mann–Whitney U test, W = 755, p = 0.674). Cultures were incubated in vented plastic storage bins on the same shelf of the growth chamber. Temperature did not vary across the shelf of the growth chamber.

A still image of each worm was taken on a stereomicroscope at 0, 18, 24, 36, and 48 h after transfer. Images were taken without removing the culture plate lids since the lids were not removable in the Parafilm group. Worms and cultures were censored for sex, death, starvation, microbial contamination, and abnormalities in movement, behavior, and observable anatomical irregularities; 2 Parafilm-treated worms were removed from the dataset prior to analysis. Most worms produced embryos around 48 h, indicating the adult stage had been reached and thus terminating data collection. The length of each worm in the still images at each time point was measured down the midline from the tip of the lips to the end of the tail using the freehand tool in ImageJ [14]. All measurements were taken blind by one observer through randomized labeling of image files by a different individual.

We calculated the change in length (Δgrowth) from 0 to 48 h as well as the slope of the best-fit linear growth curve (growth rate) through the 5 time points for each worm. The data were not normally distributed for the two growth variables, so we used Mann–Whitney U tests to compare median Δgrowth and growth rate between the Parafilm and control groups in R version 3.5.2 with α = 0.05 [15]. We also calculated the standardized treatment effect size (Cohen’s *d*) for each growth variable [16, 17].

### Results

**Fig. 2:**
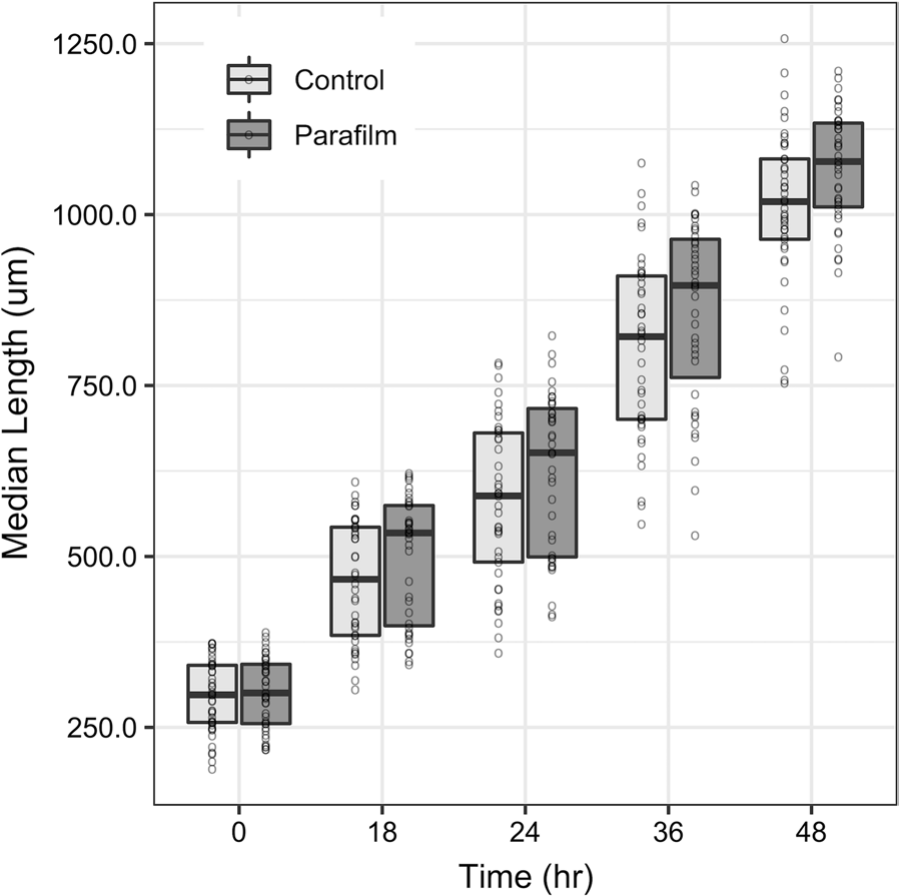
Larval growth over time. Median length (μm) of worms in each treatment group at 0, 18, 24, 36, and 48 h after L1 transfer to plates. Data are displayed as boxplots overlaid with scatterplots of individual worm lengths (open circles) at each time point. Bold line within each box shows median length, and box shows Q3 (upper quartile) and Q1 (lower quartile). n = 39 Parafilm and n = 41 control

Median larval length (Fig. 2) at each time point appeared similar with small differences between the Parafilm and control groups. However, over the 48 h period, worms in cultures wrapped with Parafilm showed significantly greater Δgrowth (Mann–Whitney U test, W = 472, p = 0.001) and a significantly faster growth rate (Mann–Whitney U test, W = 479, p = 0.002) than worms in the control group (Table 1). Furthermore, Cohen’s *d* indicated a moderate impact of Parafilm wrapping on the Δgrowth and growth rate of *C. elegans* over 48 h (Cohen’s *d* = 0.75 and *d* = 0.74 respectively).

**Table 1:** Significant differences in *C. elegans* larval growth over 48 h on Parafilm-wrapped (n = 39) and control (n = 41) plates

	Median (IQR) length (μm)	
	Parafilm	Control
ΔGrowth	764.25 (39.3)	726.35 (97.3)
Growth rate	16.43 (1.3)	15.56 (1.7)

### Discussion

This study aimed to determine if *C. elegans* larval growth is affected by wrapping NGM culture plates with Parafilm. We found that larval Δgrowth and growth rate (Table 1) were significantly greater in worms on Parafilm-wrapped cultures than worms in control cultures. Our results suggest that wrapping culture plates with Parafilm alters conditions in the internal plate environment, causing statistically detectable differences in larval growth over time compared to control conditions.

The median values for the measured variables (Δgrowth and growth rate) were rather similar between the control and Parafilm groups (Table 1), and Cohen’s *d* indicated a moderate treatment effect. The moderate treatment effect size suggests that the measured differences may not reflect biologically significant differences for all experiments, depending upon the sensitivity to which the researcher needs to quantify and compare growth among treatment groups. For example, worm length can be used as a determinate of larval stage [18] along with developmental hallmarks [19, 20]. The median Δgrowth difference between our Parafilm and control groups over 48 h (37.90 μm; Table 1) would be unlikely to cause a mislabeling of larval stage since worms grow approximately 690 μm (20 **°**C) between the first molt to egg-laying onset [18], which are the stages we observed over the course of our measurements. Conversely, the small but significant differences in median growth rate (0.87 μm/h; Table 1) that we observed from wrapping cultures with Parafilm could unintentionally lead to misinterpretation of growth rates taken at finer and more precise time scales, as growth rates are linear within a larval stage but change between larval stages [21].

Wrapping culture dishes with Parafilm is a common practice with model organisms other than *C. elegans*. For example, researchers studying the plant model *Arabidopsis thaliana* (*A. thaliana*) wrap their Petri dish cultures with Parafilm for many of the same reasons as worm researchers. Similar to our study, recent research on *A. thaliana* sought to determine if wrapping plant cultures with Parafilm affected the culture environment and plant growth [22]. In both their and our studies, there were not only changes in organismal growth as a result of Parafilm-wrapping but also reduced variability in growth among Parafilm-wrapped replicates (Fig. 1F in [22]; IQR in Table 1 and Fig. 2 in this study), suggesting that Parafilm creates standardized conditions that reduce variability in organismal responses.

Environmental factors such as diet, population density, and temperature are known to affect worm body size [23–28]. In our study, it is unlikely that diet or population density contributed to differences between worms in each group because all worms were fed the same bacterial strain ad libitum and they all experienced the same population density effects by being individually cultured. Instead, we hypothesize that *C. elegans* grown in Parafilm-wrapped cultures showed moderately significant increases in growth compared to control worms because of differences in humidity, temperature, and/or gas levels (i.e. oxygen and/or carbon dioxide).

*Caenorhabditis elegans* is capable of sensing humidity [29–32], but the direct effects of humidity on growth have not been studied to date. However, Wang et al. postulated that the amount of water content in cultures might affect the temperature in or near the agar surface [29], and the Bristol N2 wild-type strain, which was used in our study, follows the temperature-size rule by growing larger at lower temperatures [33]. Since Parafilm prevents media desiccation, Parafilm-wrapped cultures likely have differences in relative humidity and, in turn, possibly temperature near and/or above the agar–air interface where worms live compared to cultures not wrapped with Parafilm. Humidity-mediated differences in temperature could have contributed to our observed differences in growth if we consider the temperature-size rule [33], and/or humidity could have directly affected growth by undetermined mechanisms. Worms were only exposed to Parafilm or control conditions for 48 h so minimal moisture would have been lost from the control cultures, but this short exposure time could explain the similar medians measured between the Parafilm and control groups.

Recent research on *A. thaliana* cultures demonstrated that gas exchange may be affected by Parafilm wrapping. Parafilm-wrapped cultures had significantly different carbon dioxide, but not oxygen, levels compared to control cultures over short (min) and long (days) time periods [22] even though the Parafilm manufacturer [34] and distributors [35] claim that Parafilm is permeable to gases. Interestingly, previous studies suggest that starved worms, which were used in our study, are more attracted to bacterial strains that produce higher levels of carbon dioxide during respiration and this affects life history traits [36]. Taken together, it is plausible that worm and bacterial respiration caused a higher accumulation of carbon dioxide in Parafilm-wrapped compared to control cultures, resulting in increased worm attraction to and consumption of the bacteria. This increase in feeding could explain increased worm growth in the Parafilm-treated worms as compared to the control worms, especially between 0 and 18 h where we see the greatest difference in median growth between the groups, at the time when worms are initiating growth from L1 starvation (Table 1; Fig. 2).

The increased growth we observed provides evidence that there is some effect on *C. elegans* from wrapping NGM cultures with Parafilm, and supports similar effects seen with *A. thaliana* [22]. Complementary studies that measure culture conditions (e.g. humidity, temperature, oxygen and carbon dioxide) and life-history traits under Parafilm-wrapped conditions should be done in an animal model such as *C. elegans* to more comprehensively explore the effects of Parafilm on laboratory cultures. Until then, researchers of all organisms should first test for the effects that this practice could have on their measured variables. This will eliminate potential secondary effects that could inadvertently influence dependent variables, especially those for which slight differences will affect data analysis and interpretation.

## Limitations

Our study did not assess morphological or behavioral developmental hallmarks at each time point for developmental correlations to larval growth and development, nor did we evaluate development at a finer timescale. However, we observed no prominent differences in the timing of initial egg-laying between groups, suggesting worms in each group reached adulthood at approximately the same time. We also did not measure temperature, humidity, or carbon dioxide and oxygen levels inside the culture dishes to determine potential causes of differences in worm growth between Parafilm-wrapped and control cultures, and we suggest that this important issue be addressed by future studies.

## Data Availability

The datasets used and/or analyzed during the current study are available from the corresponding author on reasonable request.

## Abbreviations

NGM: Nematode Growth Media
*C.*: *Caenorhabditis*
*E.*: *Escherichia*
CGC: *Caenorhabditis* Genetics Center
Δgrowth: change in length
IQR: interquartile range
*A.*: *Arabidopsis*
